# Protein NMR Structures Refined without NOE Data

**DOI:** 10.1371/journal.pone.0108888

**Published:** 2014-10-03

**Authors:** Hyojung Ryu, Tae-Rae Kim, SeonJoo Ahn, Sunyoung Ji, Jinhyuk Lee

**Affiliations:** 1 Korean Bioinformation Center (KOBIC), Korea Research Institute of Bioscience and Biotechnology, Daejeon, The Republic of Korea; 2 Department of Bioinformatics, University of Science and Technology, Daejeon, The Republic of Korea; 3 Department of Chemistry, Seoul National University, Seoul, The Republic of Korea; University of South Florida College of Medicine, United States of America

## Abstract

The refinement of low-quality structures is an important challenge in protein structure prediction. Many studies have been conducted on protein structure refinement; the refinement of structures derived from NMR spectroscopy has been especially intensively studied. In this study, we generated flat-bottom distance potential instead of NOE data because NOE data have ambiguity and uncertainty. The potential was derived from distance information from given structures and prevented structural dislocation during the refinement process. A simulated annealing protocol was used to minimize the potential energy of the structure. The protocol was tested on 134 NMR structures in the Protein Data Bank (PDB) that also have X-ray structures. Among them, 50 structures were used as a training set to find the optimal “width” parameter in the flat-bottom distance potential functions. In the validation set (the other 84 structures), most of the 12 quality assessment scores of the refined structures were significantly improved (total score increased from 1.215 to 2.044). Moreover, the secondary structure similarity of the refined structure was improved over that of the original structure. Finally, we demonstrate that the combination of two energy potentials, statistical torsion angle potential (STAP) and the flat-bottom distance potential, can drive the refinement of NMR structures.

## Introduction

The accurate determination of three-dimensional structure is an important challenge in structural biology. Detailed and precise protein structures are essential in biological studies such as ligand docking, disease-related mutations and structure-based protein function studies, which are directly applicable to drug discovery [1]–[5]. Given the importance of protein structure, obtaining accurate and high-quality protein structures remains a major challenge. Thus, the critical assessment of techniques for protein structure prediction (CASP) competition has included a refinement category section since CASP7, called CASPR [6]. Many groups participating in the CASP developed their own approaches for structure refinement. These approaches can be broadly into two categories. The first category focuses on improving the accuracy of the energy functions to drive the lowest energy conformation to be the native structure. There exist statistically derived knowledge-based [7]–[11] and physics-based [5], [12] energy functions. In these energy functions, solvent models are also introduced implicitly and explicitly for refinement [2]. In terms of computational elapsed time, the implicit solvent model was successful in structure refinement [13]–[16]. One group added a layer of water molecules to improve protein structure quality. Because they considered minimal water molecules for refinement, the protocol was less time-consuming than conventional explicit water model [17]. Other methods involve developing sampling methods that search efficiently on the energy surface to arrive at the native state, such as the replica exchange method [13]–[15], [18], targeted MD [19], steered MD [20] and accelerated MD [21].

The protein structure refinement process has prevailed in NMR structures, especially because the quality of NMR structures is less accurate than that of X-ray crystallography structures [22], [23], which arises from the dynamic motion of proteins in solution and weak Nuclear Overhauser Effect (NOE) signal intensity. Out of necessity, several NMR structure refinement databases were introduced: REcalculated COORdinates Database (RECOORD), a database of REfined solution NMR structures (DRESS) and statistical torsion angle potential (STAP) [24]–[26]. Mao *et. al.* [27] have shown significant result of NMR refinement using both restrained and unrestrained Rosetta refinement protocol. Therefore, in this work, we performed refinements using the knowledge-based potential (STAP) developed by our group on 134 NMR structures in the Protein Data Bank (PDB). The efficiency of STAP was verified by a previous study [26]. Unlike the previous successful study on NMR structure refinement, we did not use the experimental data on the NMR structures (NOE data). The ambiguity in NOE data is one of the main problems with NMR structures [28]; this arises because the NOE signal is weak, and peak picking is difficult during structure determination/refinement processes. Instead of using such NOE restraints, in this study, we used the distance information derived from the given structure. With these distances, we created restraint energy potential, called flat-bottom distance potential (see the Methods for details). The restraints prevent structural dislocation in the refinement process. Because this approach does not require using distance restraints from experiments, it can be applied to refine both X-ray crystallographic structures and homology structures generated by the CASP competition.

## Methods

### Training and test sets

We used 1,879 structures in the PDB that have both NMR and experimental X-ray crystallography data. Because X-ray structures have higher resolution, we used them as native structures to measure backbone similarity (TM-score, RMSD and GDT-HA). Among them, we selected 134 structures with these criteria: more than 50 amino acids and an amino acid gap difference between the X-ray and NMR structures of less than 10. These 134 structures were used for testing our protocol. Among them, 50 structures were used to find the optimal width of the flat-bottom distance potential (see the next section), and 84 structures were used as a test set to benchmark our method. The information of used structures (NMR and the corresponding X-ray) is tabulated in Tables S1 (training set) and S2 (test set). The table has the PDB ID, chain name, and number of amino acid, secondary structure diversity, and resolution of X-ray structure.

### STAP and flat-bottom distance potential for structure refinement

Two new energy potentials are introduced for the refinement: STAP and flat-bottom distance potential. STAP is focused on torsion angle and is a grid type knowledge-based energy function for individually collected torsion angle populations of φ-ψ, φ-χ_1_, ψ- χ_1_ and χ_1_-χ_2_, where each torsion angle combination set consists of functions of 21 amino acids (20 normal amino acids and pre-proline). The torsion angle populations are obtained from high-resolution X-ray structures under 2.0 Å. The efficacy of STAP was demonstrated in the earlier research, such as homology modeling and NMR structure refinement [7], [26].

**Figure 1:**
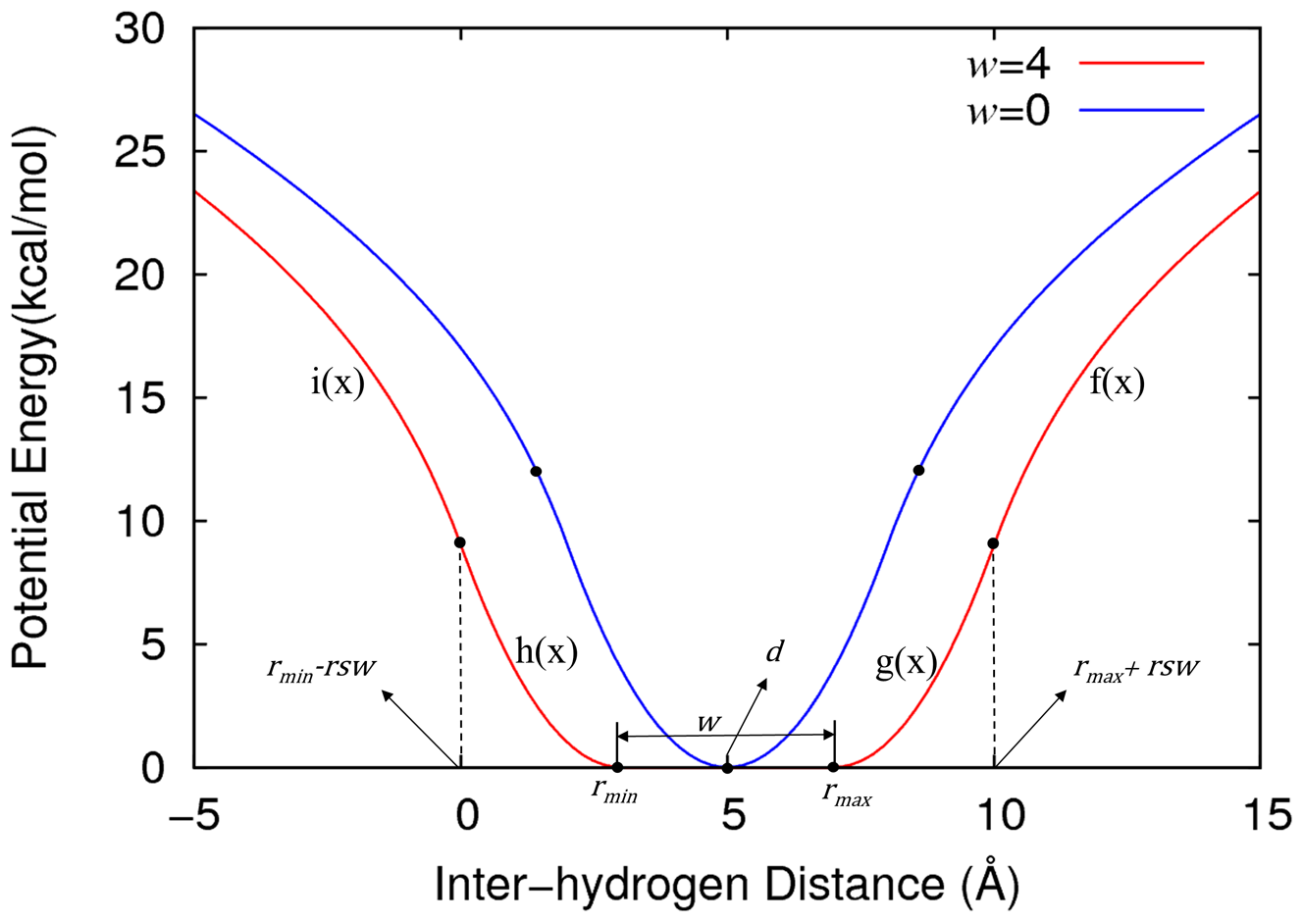
Two flat-bottom distance potential functions: the same equilibrium distance (*d*) of 5 Å and two flat-bottom widths (*w*), 0 (blue) and 4 (red line). The used parameters are defined in Method section.



A flat-bottom distance potential function (originally introduced in the reference [29]) is shown in Fig. 1. The potential function is composed of two main variables: the equilibrium distance (*d*) of two interacting hydrogen atoms and the flat bottom width (*w*). All inter-hydrogen distances are obtained from the given original structure. From these interactions, we choose the distances of two atoms below 7 Å (cutoff distance). Although the NMR experiments consider 6 Å as a long-range distance, we heuristically select the cutoff distance as 7 Å. From these distances, the equilibrium distance for the flat-bottom distance potential are calculated by two methods (r6 summation and shortest distance; described in the next section). Finally, the flat-bottom potential functions are generated with the equilibrium distance and various flat-bottom widths from 0 to 10 Å at intervals of 1 Å. The flat-bottom distance potential (*U_fb_*) is defined as

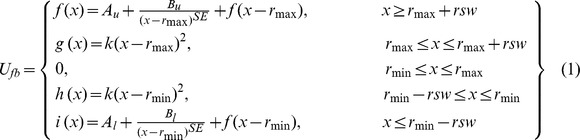
where *f(x)* and *i(x)* functions are soft asymptote functions, and *g(x)* and *h(x)* functions are quadratic functions (Fig. 1). The soft asymptote functions are introduced to prevent large atomic force occurring at long inter-hydrogen distance during a simulation. The *r*_min_ and *r*_max_ are defined in terms of *d* and *w*, *r*_min_  =  *d-w*/2 and *r*_max_  =  *d+w*/2. To make their functions to be smooth, *i.e.* continuity and derivative continuity, *A_u_, B_u_, B_l_,* and *A_l_* are obtained in terms of four parameters (*SE*, *f*, *k*, and *rsw*) as followings.



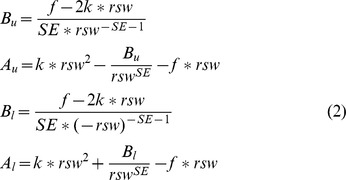
where *SE* is exponent of the soft asymptote that is usually set to 1, and *f* is slope of the asymptotic function (defined value is 1). The *k* is a force constant for the quadratic function that is set to 1/2, and *rsw* is function range of quadratic function and defined as a value of 3.

### Two computational experiments

Two computational experiments (*S1*: r6 summation and *S2*: shortest distance) were performed. The *S1* experiment used the equilibrium distance of the flat-bottom distance potential generated by the r6 summation method based on the equation, 
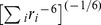
, where *r* is the distance between two interacting atoms, and *i* is the index of the interaction pairs [28]. For example, there are six interaction pairs between the three β hydrogen atoms attached to C_β_ and the two γ hydrogen atoms on C_γ_. The inter-distance in the r6 summation was calculated using the equation above. The *S2* experiment did not take into account the all-atom pairs and considered only the distance information of the shortest interaction atom pair.

### Simulated annealing (SA) protocol

To refine the structures, simulated annealing was used to minimize the target energy (*E_total_*; Eq. 3), which consists of the default CHARMM energy [30] with EEF1.1 solvation energy (all hydrogen effective energy functions [31] included in the CHARMM parameters) (*E_CHARMM_*), STAP (*E_STAP_*), and flat-bottom distance potential (*E_flat_*). The *E_flat_* was scaled by a factor of 10.0.
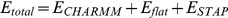
(3)


The refinement protocol that was used is as follows: (i) the system is minimized and heated from 100 to 500 K using 1,600 molecular dynamics steps; (ii) three annealing steps (2,000, 5,000, and 10,000 steps) are performed at 500 K with molecular dynamics; (iii) a cool-down to 25 K runs for 4,000 steps; and (iv) a short minimization is performed with 100 steps. All of the simulations were executed using CHARMM [32].

### Quality assessment scores

The quality of the structures obtained after the refinement simulations was considered by various quality assessment scores: backbone similarity (assessed by TM-score [33]), number of NOE violations (NOE) [34], two protein energy scores measured by nDOPE (normalized DOPE) [35], dDFIRE (dipolar Distance-scaled, Finite-Ideal gas Reference) [36], clash score of atoms (measured by Molprobity (clash) [37]); two percentages (MolRama and ProRama) of favorable Ramachandran (by Molprobity [37] and PROCHECK [38]), and five normalized scores (pack1, pack2, WhatRama, Rotamer, and backbone; by WHAT_CHECK [39]). Because the TM-score is independent of protein-size, it is used for default backbone similarity. The NOE violation was measured using known experimental NOE data obtained from BMRB (Biological Magnetic Resonance Bank) [40]. Hereafter, we define a “protein-like” score excluding two scores: TM-score and NOE violations. Because the various scores are measured from a structure, we calculate one normalized score (total score): “good” and “bad” values and the weight for each score are tabulated in Table S3. The assigned weights was used in the previous study [7]. The highest total score indicates the best structure and is used to find the optimal width.

## Results and Discussion

### Total score change as a function of flat-bottom width

Two computational simulations (*S1* and *S2*) were performed for NMR structure refinement. In this section, 396 NMR structures that were randomly extracted from the STAP DB [26] were used. Because there were no corresponding X-ray structures, TM-scores were measured using their own NMR structures. In the *S1* simulation, total score (weighted summation of various scores) changes were observed from the three annealing steps (2,000, 5,000, and 10,000 steps) as shown in Tables S4, S5, and S6, respectively. Although homology modeling structures in the previous study [41] were gradually improved as the number of annealing steps was increased, in this work there are no great differences among the total scores from each of the annealing steps (as shown in Fig. S1), indicating that the annealing step does not affect these refinement simulations and that the annealing time of 2,000 steps is suitable for NMR structure refinement.

The TM-score and NOE violations in the *S1* simulation show marginal change as the width of the flat-bottom potential increases from 0.0 to 10.0 (Table S4). Although we used a width of 0.0 Å, which means that the flat bottom has no flat region and the structure maintains its original structure, the TM-score decreased to 0.788 (note that the reference structure is the own NMR structure) and the NOE violations increased to 0.539. This abnormal tendency could be caused by using the r6 summation to generate the equilibrium distance. The r6 summation is generally used in NMR structure calculation because of existing indistinguishable hydrogen atoms, such as two or three hydrogen atoms attached to a carbon atom. Many distance combinations are available that satisfy the given equilibrium distance. These distance combinations generate diverse conformations that deviate from the values of the original structure. Because there were significant changes in the TM-score and NOE violations at the width of 0.0 Å (Table S4), another simulation (*S2*) was performed. The *S2* simulation used the shortest distance from atom interaction pairs for the equilibrium distance. The TM-score and NOE violations changed gradually rather than suddenly (Table S7). The best total score (1.972) was located at the width of 2.0 Å, while the best total score in the *S1* simulation was 1.624 at the width of 6.0 Å (Table S4). Note that this protein set used the original NMR structure for reference structure of TM-score. Training set in the next section will use the corresponding X-ray structure for a reference. Consequently, because the *S2* simulation produced better total scores for refinement than did the *S1* simulation, the *S2* simulation protocol (2,000 annealing step and shortest distance) was used for further simulations.

### Optimization of flat-bottom width parameter for the training set

**Figure 2:**
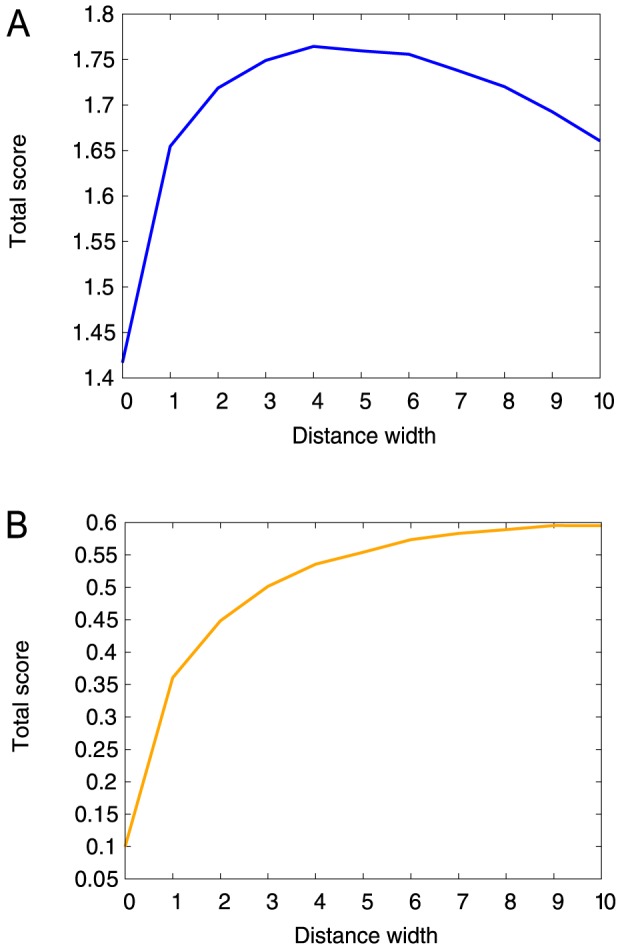
Total score change in the training set (A) considering all quality assessment scores (TM-score, NOE violations and “protein-like” scores), and (B) considering only “protein-like” scores. The optimal width for (A) is 4.0 Å, while it is gradually improved in (B).

The previous section showed that the best total score changed with the width of flat-bottom distance potential. In this section, we found the optimal width parameter of the potential at which the total score is maximized. In the total score, the TM-score was calculated using the corresponding X-ray structure as a reference. The width parameters were changed from 0.0 to 10.0 Å with an interval of 1.0 Å (11 parameters in total). As the width was increased, NOE violations gradually deteriorated, while the TM-score achieved its best score at the width of 4.0 Å (Fig. 2 and Table S8). The “protein-like” score gradually improved. In detail, the WHAT_CHECK Ramachandran plot appearance of “protein-like” score improved dramatically from −2.224 (width of 0.0 Å) to 2.060 (width of 10.0 Å). The clash score also improved from 14.07 to 0.13, and other “protein-like” scores were generally improved as the distance width increased (Fig. 2B and Table S8). In summary, the TM-score and NOE violations results were better at small widths, whereas “protein-like” scores were better at large widths. Because the total score is a weighted summation of all of the scores used (TM-score, NOE violation and “protein-like” scores), the best total score was located in the middle, at a width of 4.0 Å (Fig. 2A). Thus, the width of 4.0 Å is called the optimal width. In the next section, NMR refinement simulations were performed on the validation set using the optimal width.

### Test refinement simulations with the optimal width

Refinement simulations were run for 84 NMR structures to investigate how the protein structures were improved with the flat-bottom distance potential and without considering any experimental NMR distance information. As shown in Table 1 and Fig. S2, most quality assessment scores were improved over those of the original NMR structure. In particular, the clash score was clearly decreased from 53.68 to 0.35, and the Ramachandran plot appearance was improved a great deal. It is known from previous studies that the knowledge-based potential that was used, STAP, greatly impacts Ramachandran-relevant scores. Energy scores, such as nDOPE and dDFIRE scores, were stabilized, and the TM-score also improved from 0.782 to 0.792 (negligible but 1% improved in backbone accuracy). The RMSD also improved over the original structure (from 3.113 Å to 3.076 Å) and GDT-TS and GDT-HA (other backbone similarity indicators) increased from 0.757 to 0.773, from 0.562 to 0.592, respectively. Visual inspections of individual target structures will be described in the next section. Unfortunately, NOE violation distance increased from 0.104 to 0.335 Å. Note that this refinement did not use the experimental distance information (NOE data), and the NOE violation of 0.335 Å is not a bad result because the experimental NOE distance measurement has an error of distance [28] of approximately 0.5∼1.0 Å. Given that there were 12 (our refined structure) and 9 (original NMR structure) instances of the number of violated NOE distances over 2.0 Å, the results indicate that most NOE violations are located below 1.0 Å, and a difference of 3 violations is so small as to be negligible.

**Table 1:** Comparison of refined structures using the optimal width with original NMR structures.

Score	TM-score^a^	NOE Violations (0.5/1.0/2.0)^c^	nDOPE	dDFIRE	Clash	Rama (MOL)^d^	Rama (PRO)^e^	1^st^ Packing	2^nd^ Packing	Rama (WHAT)^f^	Rotamer	Backbone	Total score	RMSD^a^	GDT-TS^a^	GDT-HA^a^
Refined structures (Optimal width)	**0.792** b	0.335 (87/41/12)	**−1.3094**	**−227.89**	**0.35**	**95.10**	**90. 43**	**−2.060**	**−1.321**	**1.714**	**1.302**	**−0.804**	**1.899**	**3.076**	**0.773**	**0.592**
Original NMR Structures	0.782	**0.104 (42/25/9)**	−0.8232	−206.894	53.68	81.81	74.71	−2.511	−2.424	−4.667	−5.59	−1.020	1.081	3.113	0.757	0.562

a Four Structural similarities are measured by TM-score program (reference atom: C_α_): TM-score, RMSD, GDT-TS, and GDT-HA. The used reference structure is X-ray structure (Table S2).

b Bold font numbers indicate better scores.

c Number of violated NOE distances over 0.5, 1.0, and 2.0 Å. The NOE violations are measured with the experimental NOE data obtained from BMRB (Biological Magnetic Resonance Bank).

d Ramachandran appearance measured using MolProbity.

e Ramachandran appearance measured using PROCHECK.

f Ramachandran appearance measured using WHAT_CHECK.

### NMR refinement simulations for the entire width range

**Figure 3:**
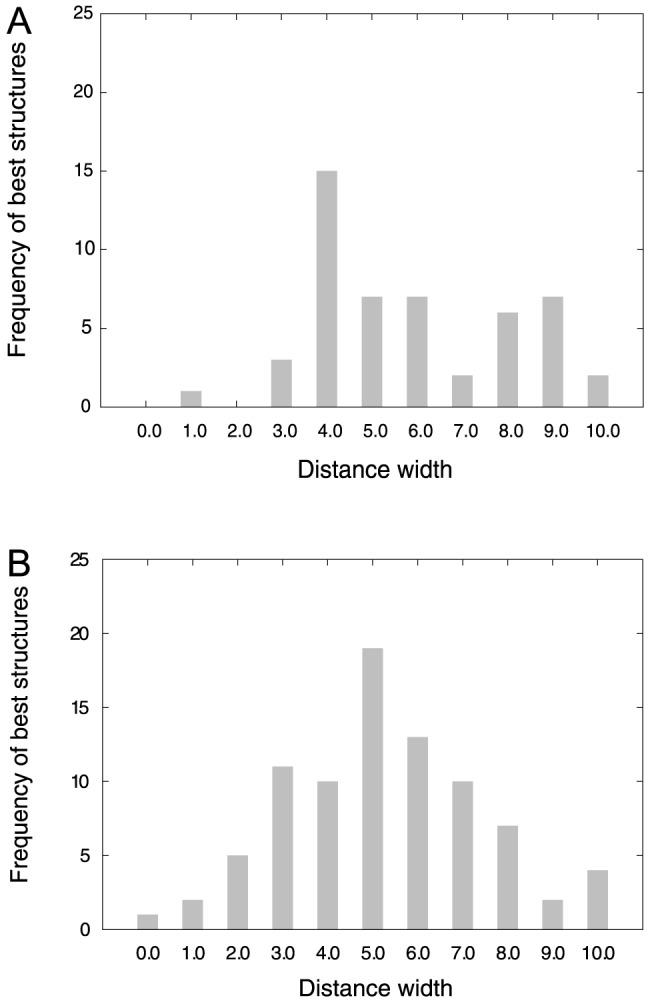
Frequency of the best structures in the (A) training and (B) test sets as a function of flat-bottom width from 0 to 10 Å.

**Figure 4:**
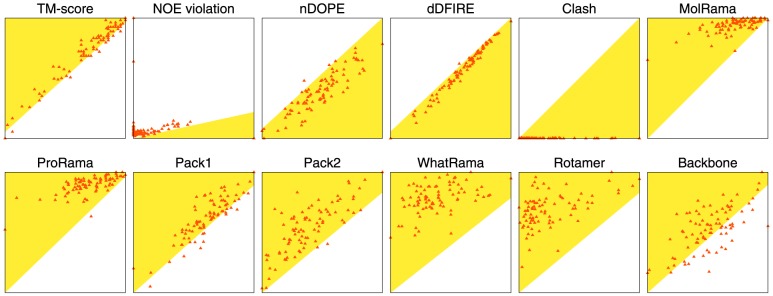
Comparison of quality assessment scores for each of the best structures. The shaded yellow color indicates the region where the best refined structures (Y-axis) are better than the original structures (X-axis).

The previous section demonstrated that the refinement simulations performed at the optimal width obtained better scores than the original NMR structures. In this section, these simulations were run for the entire range of widths from 0.0 to 10.0 Å. The best structure obtained for each target was not always at the optimal width (4.0 Å). Fig. 3 shows the frequency of the best structures as a function of width. The largest frequencies for the training set (50 structures) and the test set (84 structures) were at widths of 4.0 and 5.0 Å, respectively. Note that some NMR target structures had their best total scores anywhere from 0.0 to 10.0 Å. Quality assessment scores were tabulated using the best structure (Table 2). The TM-score improved substantially from 0.795 to 0.820 (2.5% increase), and protein-quality scores were also improved over those obtained at the optimal width. As comparison results [27], [42], a recent procedure for NMR refinement with Rosetta method showed that average GDT-TS score of 39 NMR structures was improved by 2.5% (using experimental NMR restraints) and 0.4% (without NMR restraints) [27]. Our GDT-TS score is improved by 4.7% (from 0.757 to 0.804). Thus, our refinement protocol is comparable with the refinement method (Rosetta method). As shown in Fig. 4, most structures were distributed in the refined region (shaded by yellow). Although NOE violation distances were not improved over those in the original structures, the number of violated NOE distances decreased to 35/21/8 and arrived at similar values to those of the original NMR structures (35/20/8). This result indicates that most violated NOEs are located from 0 to 0.5 Å.

**Table 2:** Comparison of refined structures using best width with original NMR structures^a^.

Score	TM-score	NOE Violations (0.5/1.0/2.0)	nDOPE	dDFIRE	Clash	Rama (MOL)	Rama (PRO)	1^st^ Packing	2^nd^ Packing	Rama (WHAT)	Rotamer	Backbone	Total	RMSD	GDT-TS	GDT-HA
Refined structures (Best width)	**0.820**	0.321 (35/21/8)	**−1.4213**	**−232.959**	**0.19**	**96.34**	**92.0**	**−1.704**	**−0.861**	**2.352**	**2.247**	**−0.472**	**2.044**	**2.793**	**0.804**	**0.629**
Original NMR structures	0.795	**0.09 (**35**/20/**8**)**	−0.8838	−209.13	47.22	83.78	76.5	−2.369	−2.312	−4.423	−5.221	−0.854	1.215	3.113	0.757	0.562

a See the footnotes in Table 1.

**Figure 5:**
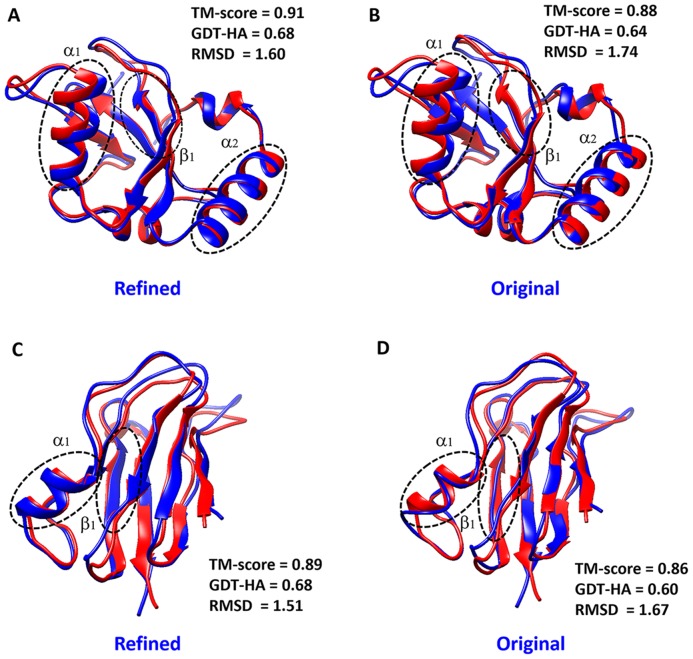
Two examples of our refinement on 1KOT and 1FA4. The structures are drawn as cartoons using Chimera [47]. The refined and original structures (blue color cartoons) are superimposed with respect to their reference structures: X-ray structures (red color cartoons; PDB ID: 3D32 for sub-figures A and B, and PDB ID: 2CJ3 for sub-figures C and D). Dashed circles in the structures represent the apparent secondary structure regions improved by our method. The backbone accuracies with regard to the reference structure are calculated with the TM-score, the GDT-HA score, and the RMSD, where those scores are measured using the TM-score program.

Here, we describe two illustrative examples that showed the best performance in refinements using a width of 4.0 Å (Fig. 5). The β-strand region in the refined structure (PDB ID: 1KOT) was well-created (β1 region), and the helix (α1 and α2 regions in Figs. 5A and B) and loop regions were well-oriented to fit the native structure. The backbone accuracy of the refined structure increased from 0.88 to 0.91 (TM-score), from 0.64 to 0.68 (GDT-HA), and the RMSD decreased from 1.74 to 1.60 Å. As a second example, in structure (1FA4), the α helix was well-generated in the refined structure (α1 in Figs. 5C and D). Moreover, we see that the coil region in the original structure was significantly improved in the β-strand in the refined structure (β1 in Figs. 5C and D). The TM-score, the GDT-HA score and the RMSD of the refined structure were also better than those of the original structure.

**Figure 6:**
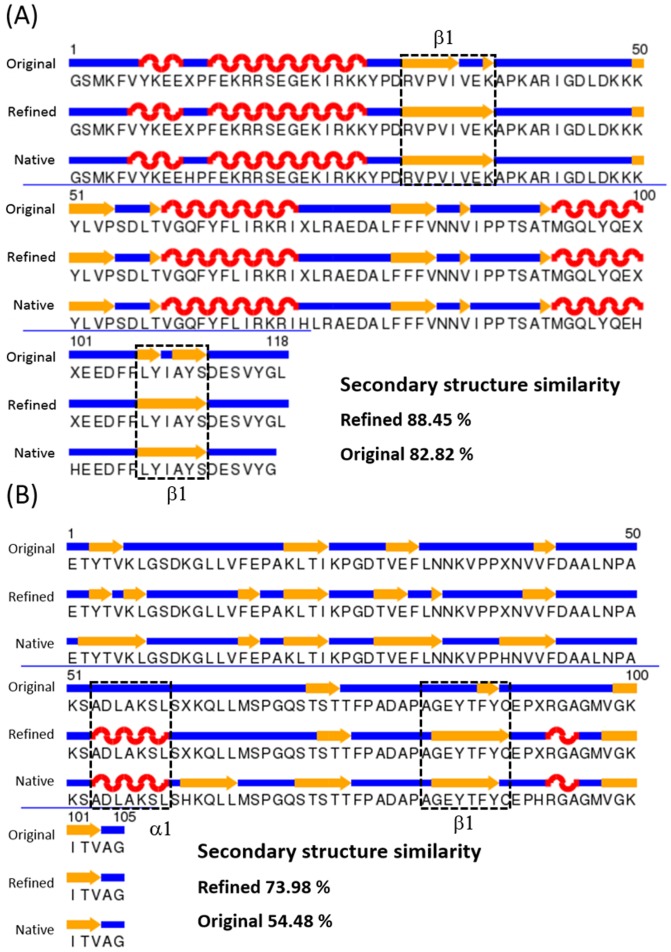
Secondary structure schemes of three conformations (original, refined, and native (X-ray)) of PDB (A) 1KOT and (B) 1FA4. The black dashed lines indicate the refined regions.

In Fig. 5, we see that the secondary structures were improved in the refined structures. In particular, β-strand regions of the refined structures were well generated. Thus, we compared the similarity of the secondary structures of the refined/original structures with that of the native structures (X-ray). The secondary structures were evaluated with DSSP [43]. Overall secondary structure similarity (α,β and coil state) between the X-ray and refined structures is 76.78%, which is better than that of the original structure (73.15%). In particular, the individual similarity (the match percentages) of α, β and coil regions increased from 80.52% to 82.66%, 75.22% to 81.31% and 25.71% to 26.04%, respectively. The β region was much more improved than the others, indicating that our protocol drives proteins to generate secondary structures. For example, β1 (residues 30–37, 107–112) in the refined structure of 1KOT was well generated, and a high similarity of 88.45% can be observed (Fig. 6A). The secondary structures of 1FA4 α1 (residues 53–59) and β1 (residues 83–89)) look similar to those of the X-ray structure (Fig. 6B). Furthermore, the similarity of the secondary structure was greatly increased, from 54.58% to 73.98%.

### Comparison with re-refinement method

Protein structures derived from NMR experiments undergo a refinement step before their structures are deposited in the PDB. The refinement tools that are mainly used are X-PLOR [44], AMBER [45], RECOORD [25], and CNS [46]. Among them, we compared the quality of structures refined by AMBER/RECOORD with those of our refined structures that were refined using the optimal width 4.0. We found 23 structures from our target structure list that were re-refined by AMBER or RECOORD (Table S9). The quality of the structures refined by our method is better than that of AMBER/RECOORD-refined structures (Table 3). Because this comparison set does not have the corresponding X-ray structure, TM-score could not be measured and compared. The result has a most significant improvement on the “protein-like scores”; especially Ramachandran plot appearance score and the clash score were greatly improved, similar to the test set results. Although NOE violation of the refined structure increased by 0.117 Å than that of the re-refinement method, the other quality assessment scores are significantly improved. Thus, our method is comparable to the re-refinement method (AMBER/RECOORD).

**Table 3:** Comparison between our refinement and the re-refinement structures^a^^,^ b.

Score	Ours	AMBER & RECOORD
NOE violation	0.464	**0.347**
nDOPE	**−1.1923**	−1.0911
dDFIRE	**−186.92**	−177.856
Clash	**0.42**	6.31
Rama (MOL)	**95.12**	82.94
Rama (PRO)	**90.36**	74.26
1^st^ packing	**−3.018**	−3.16
2^nd^ packing	**−1.977**	−2.326
Rama (WHAT)	**1.193**	−4.180
Rotamer	**0.469**	−5.037
Backbone	**−1.182**	−1.306
Total	**1.0171**	0.6870

a See the footnotes in Table 1.

b A total of 24 NMR structures were used (lists are in Table S9). Because no corresponding X-ray structures exist, the TM-score cannot be measured.

## Conclusions

Many protein structure refinement approaches are performed using experimental structural data, and the results are good. In the previous NMR structure refinement approach using STAP, improved results were successfully shown. However, NOE data of NMR structures are ambiguous, and solving this ambiguity is a major problem in NMR structure determination. In this work, we did not use any experimental information (NOE distance data). Instead, we introduced a flat-bottom distance potential with the equilibrium distance information from the structure; this constraint largely prevents deviation from the current state of the original structure. The optimal width parameter was obtained in this study, and the results were improved from those of the original structure. Consequently, most of the various quality assessment scores were improved. Because this simulation does not use any experimental data and although the results for the NOE violation score were slightly increased, this refinement protocol is useful for the NMR protein structure community.

## Supporting Information

Figure S1 Total score changes of three simulations (*S1_2K*, *S1_5K*, and *S1_10K*) as a function of distance width.
(DOCX)

Figure S2 Comparison of quality assessment scores of whole structures. Shaded green color indicates the region where the refined structures (Y-axis) are better than the original structures (X-axis).
(DOCX)

Table S1 PDB list with corresponding X-ray structures (training set).
(DOCX)

Table S2 PDB list with corresponding X-ray structures (test set).
(DOCX)

Table S3 Various scores and their weights for the normalized score.
(DOCX)

Table S4 Quality assessment scores and total score in *S1* 2,000 step.
(DOCX)

Table S5 Quality assessment scores and total score in *S1* 5,000 step.
(DOCX)

Table S6 Quality assessment scores and total score in *S1* 10,000 step.
(DOCX)

Table S7 Quality assessment scores and total score in *S2*.
(DOCX)

Table S8 Quality assessment scores in 50 optimization set.
(DOCX)

Table S9 PDB list of AMBER or RECOORD comparison set.
(DOCX)
