# Supplementary figures and images for: Protein NMR Structures Refined without NOE Data
Source: PLoS One. 2014 Oct 3;9(10):e108888. doi: 10.1371/journal.pone.0108888 (PMC4184813; doi:10.1371/journal.pone.0108888)

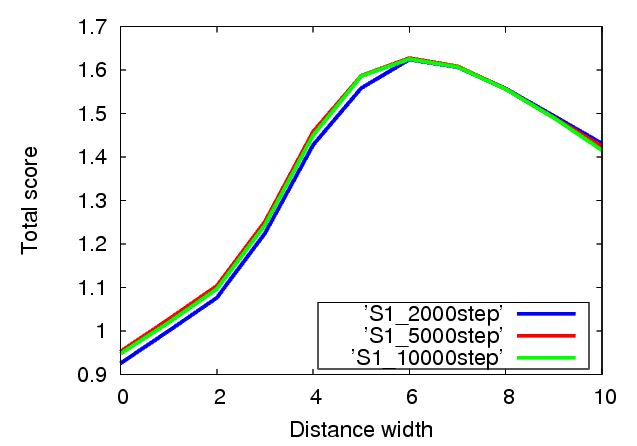


Figure S1. Total score changes of three simulations (*S1_2K*, *S1_5K*, and *S1_10K*) as a function of distance width.

Supplement: Figure S1 — Total score changes of three simulations (S1_2K, S1_5K, and S1_10K) as a function of distance width. (DOCX) [file pone.0108888.s001.docx]
