# Supplementary material for: Protein NMR Structures Refined without NOE Data
Source: PLoS One. 2014 Oct 3;9(10):e108888. doi: 10.1371/journal.pone.0108888 (PMC4184813; doi:10.1371/journal.pone.0108888)
Supplement: Figure S2 — Comparison of quality assessment scores of whole structures. Shaded green color indicates the region where the refined structures (Y-axis) are better than the original structures (X-axis). (DOCX) [file pone.0108888.s002.docx]

Figure S2. Comparison of quality assessment scores of whole structures. Shaded green color indicates the region where the refined structures (Y-axis) are better than the original structures (X-axis).
