# Supplementary material for: Protein NMR Structures Refined without NOE Data
Source: PLoS One. 2014 Oct 3;9(10):e108888. doi: 10.1371/journal.pone.0108888 (PMC4184813; doi:10.1371/journal.pone.0108888)
Supplement: Table S1 — PDB list with corresponding X-ray structures (training set). (DOCX) [file pone.0108888.s003.docx]

Table S1. PDB list with corresponding X-ray structures (training set)

| PDB ID  **(NMR)** | Chain | Number of amino acid | Secondary structure diversity (%) | | | PDB ID  **(X-ray)** | Chain | Number of amino acid | Secondary structure diversity (%) | | | Resolution (Å) |
| --- | --- | --- | --- | --- | --- | --- | --- | --- | --- | --- | --- | --- |
|  |  |  | Helix | Beta | Coil |  |  |  | Helix | Beta | Coil |  |
| 1A24 | A | 189 | 48.41 | 9.26 | 42.33 | 1FVK | A | 189 | 52.13 | 11.17 | 36.70 | 1.7 |
| 1BEG | A | 98 | 57.79 | 6.01 | 36.20 | 1BEO | A | 98 | 61.22 | 6.12 | 32.65 | 2.2 |
| 1BF8 | A | 205 | 2.07 | 41.73 | 56.20 | 3BWU | C | 205 | 3.00 | 52.00 | 45.00 | 1.76 |
| 1BFY | A | 54 | 0.56 | 17.78 | 81.67 | 1IRO | A | 54 | 16.98 | 22.64 | 60.38 | 1.1 |
| 1CK2 | A | 104 | 38.46 | 19.23 | 42.31 | 1NMU | B | 104 | 46.15 | 18.27 | 35.58 | 2.31 |
| 1CN7 | A | 104 | 40.67 | 18.27 | 41.06 | 1NMU | B | 104 | 46.15 | 18.27 | 35.58 | 2.31 |
| 1CWX | A | 44 | 2.27 | 0.00 | 97.73 | 1XF5 | P | 44 | 0.00 | 0.00 | 100.00 | 2.6 |
| 1E8L | A | 129 | 34.36 | 5.95 | 59.69 | 2VB1 | A | 129 | 40.31 | 10.85 | 48.84 | 0.65 |
| 1F2G | A | 58 | 15.86 | 13.79 | 70.34 | 1FXD | A | 58 | 24.56 | 21.05 | 54.39 | 1.7 |
| 1FQQ | A | 41 | 9.76 | 28.54 | 61.71 | 1FD3 | A | 41 | 14.63 | 31.71 | 53.66 | 1.35 |
| 1G6J | A | 76 | 19.20 | 32.69 | 48.11 | 3NHE | B | 76 | 23.68 | 30.26 | 46.05 | 1.26 |
| 1GH1 | A | 90 | 57.48 | 0.00 | 42.52 | 1BWO | A | 90 | 62.22 | 0.00 | 37.78 | 2.1 |
| 1IGL | A | 67 | 29.55 | 3.28 | 67.16 | 3KR3 | D | 67 | 55.00 | 3.33 | 41.67 | 2.2 |
| 1IMQ | A | 86 | 52.33 | 0.00 | 47.67 | 2VLN | A | 86 | 61.25 | 2.50 | 36.25 | 1.6 |
| 1JAS | A | 152 | 35.76 | 16.84 | 47.40 | 2YB6 | A | 152 | 35.33 | 20.00 | 44.67 | 1.5 |
| 1JNJ | A | 100 | 0.00 | 34.60 | 65.40 | 1K5N | B | 100 | 0.00 | 49.00 | 51.00 | 1.09 |
| 1K0S | A | 151 | 9.60 | 39.60 | 50.79 | 2CH4 | W | 151 | 10.07 | 42.45 | 47.48 | 3.5 |

| PDB ID  **(NMR)** | Chain | Number of amino acid | Secondary structure diversity (%) | | | PDB ID  **(X-ray)** | Chain | Number of amino acid | Secondary structure diversity (%) | | | Resolution (Å) |
| --- | --- | --- | --- | --- | --- | --- | --- | --- | --- | --- | --- | --- |
|  |  |  | Helix | Beta | Coil |  |  |  | Helix | Beta | Coil |  |
| 1K0X | A | 108 | 4.91 | 38.56 | 56.53 | 1I1J | A | 108 | 5.66 | 39.62 | 54.72 | 1.39 |
| 1KUN | A | 58 | 18.88 | 23.19 | 57.93 | 1KTH | A | 58 | 20.69 | 29.31 | 50.00 | 0.95 |
| 1OJG | A | 136 | 35.78 | 20.52 | 43.70 | 3BY8 | A | 142 | 42.86 | 24.81 | 32.33 | 1.45 |
| 1POQ | A | 118 | 0.25 | 35.21 | 64.53 | 1PM4 | A | 119 | 2.56 | 50.43 | 47.01 | 1.75 |
| 1QKH | A | 92 | 23.03 | 19.11 | 57.86 | 1N32 | S | 92 | 23.75 | 16.25 | 60.00 | 3 |
| 1QQI | A | 104 | 36.54 | 23.08 | 40.38 | 1GXQ | A | 106 | 39.05 | 21.90 | 39.05 | 2 |
| 1QQV | A | 67 | 41.79 | 2.99 | 55.22 | 1YU5 | X | 67 | 52.24 | 0.00 | 47.76 | 1.4 |
| 1RZW | A | 123 | 37.40 | 19.51 | 43.09 | 3ERJ | A | 123 | 42.24 | 23.28 | 34.48 | 1.8 |
| 1SB6 | A | 64 | 34.38 | 21.64 | 43.98 | 2XMJ | A | 64 | 34.92 | 20.63 | 44.44 | 1.08 |
| 1SDF | A | 67 | 16.42 | 26.87 | 56.72 | 1QG7 | A | 67 | 20.97 | 35.48 | 43.55 | 2 |
| 1SE7 | A | 83 | 52.32 | 1.03 | 46.64 | 2IDO | B | 83 | 70.67 | 0.00 | 29.33 | 2.1 |
| 1SLJ | A | 96 | 7.81 | 30.94 | 61.25 | 1SMX | A | 96 | 13.79 | 35.63 | 50.57 | 1.8 |
| 1SSU | A | 51 | 13.14 | 0.20 | 86.67 | 1OC0 | B | 51 | 21.62 | 5.41 | 72.97 | 2.28 |
| 1TOF | A | 112 | 40.18 | 22.40 | 37.42 | 1EP7 | A | 112 | 43.75 | 25.89 | 30.36 | 2.1 |
| 1V49 | A | 120 | 30.83 | 20.00 | 49.17 | 2ZJD | A | 130 | 36.36 | 23.97 | 39.67 | 1.56 |
| 1XFR | A | 128 | 66.41 | 0.78 | 32.81 | 1DQE | A | 137 | 61.31 | 1.46 | 37.23 | 1.8 |
| 1XPV | A | 78 | 37.12 | 15.26 | 47.63 | 1TTZ | A | 87 | 37.84 | 21.62 | 40.54 | 2.11 |
| 1XPW | A | 153 | 3.95 | 51.29 | 44.76 | 1TVG | A | 153 | 5.88 | 53.68 | 40.44 | 1.6 |
| 2EVN | A | 103 | 28.59 | 24.03 | 47.38 | 2IL4 | A | 103 | 36.67 | 30.00 | 33.33 | 2.05 |
| 2GPF | A | 72 | 18.26 | 26.81 | 54.93 | 2PST | X | 74 | 29.51 | 31.15 | 39.34 | 1.8 |
| 2I9H | A | 103 | 24.51 | 18.25 | 57.23 | 3F3Q | A | 109 | 36.70 | 24.77 | 38.53 | 1.76 |

| PDB ID  **(NMR)** | Chain | Number of amino acid | Secondary structure diversity (%) | | | PDB ID  **(X-ray)** | Chain | Number of amino acid | Secondary structure diversity (%) | | | Resolution (Å) |
| --- | --- | --- | --- | --- | --- | --- | --- | --- | --- | --- | --- | --- |
|  |  |  | Helix | Beta | Coil |  |  |  | Helix | Beta | Coil |  |
| 2JN0 | A | 61 | 3.00 | 50.10 | 46.90 | 3FIF | A | 61 | 5.77 | 26.92 | 67.31 | 2.7 |
| 2JPS | A | 105 | 71.75 | 0.00 | 28.25 | 3LCN | A | 105 | 70.83 | 0.00 | 29.17 | 2 |
| 2JZ2 | A | 66 | 0.68 | 45.00 | 54.32 | 3C4S | A | 66 | 5.26 | 56.14 | 38.60 | 1.7 |
| 2JZT | A | 142 | 28.73 | 15.21 | 56.06 | 2ES7 | A | 142 | 27.72 | 18.81 | 53.47 | 2.8 |
| 2K07 | A | 175 | 36.60 | 16.17 | 47.23 | 3EVX | A | 175 | 36.54 | 17.31 | 46.15 | 2.54 |
| 2K11 | A | 127 | 18.11 | 29.92 | 51.97 | 1Z7X | X | 129 | 20.63 | 36.51 | 42.86 | 1.95 |
| 2K2X | A | 75 | 12.60 | 7.67 | 79.73 | 1ZLH | B | 75 | 31.08 | 22.97 | 45.95 | 1.7 |
| 2K8Y | A | 150 | 46.40 | 16.00 | 37.60 | 3ENH | C | 150 | 41.30 | 12.32 | 46.38 | 3.6 |
| 2KI3 | A | 126 | 8.25 | 36.59 | 55.16 | 3NI6 | A | 126 | 12.10 | 36.29 | 51.61 | 1.42 |
| 2KJV | A | 101 | 28.32 | 20.30 | 51.39 | 1VOQ | F | 101 | 20.79 | 7.92 | 71.29 | 11.5 |
| 2KM0 | A | 74 | 5.27 | 32.91 | 61.82 | 3DSO | A | 74 | 4.55 | 45.45 | 50.00 | 1.55 |
| 2KO3 | A | 76 | 22.43 | 33.62 | 43.95 | 1NDD | A | 76 | 25.68 | 33.78 | 40.54 | 1.6 |
