# Supplementary material for: Protein NMR Structures Refined without NOE Data
Source: PLoS One. 2014 Oct 3;9(10):e108888. doi: 10.1371/journal.pone.0108888 (PMC4184813; doi:10.1371/journal.pone.0108888)
Supplement: Table S2 — PDB list with corresponding X-ray structures (test set). (DOCX) [file pone.0108888.s004.docx]

Table S2. PDB list with corresponding X-ray structures (test set)

| PDB ID  **(NMR)** | Chain | Number of amino acid | Secondary structure diversity (%) | | | | | PDB ID  **(X-ray)** | Chain | Number of amino acid | Secondary structure diversity (%) | | | | | Resolution (Å) |
| --- | --- | --- | --- | --- | --- | --- | --- | --- | --- | --- | --- | --- | --- | --- | --- | --- |
|  |  |  | Helix | | Beta | | Coil |  |  |  | Helix | Beta | | Coil | |  |
| 1AA9 | A | 171 | 34.71 | | 19.41 | | 45.88 | 6Q21 | A | 171 | 30.41 | 28.07 | | 41.52 | | 1.95 |
| 1AB7 | A | 89 | 40.95 | | 15.15 | | 43.90 | 1BRS | D | 89 | 50.57 | 18.39 | | 31.03 | | 2 |
| 1B75 | A | 94 | 11.38 | | 38.40 | | 50.21 | 1DFU | P | 94 | 21.28 | 43.62 | | 35.11 | | 1.8 |
| 1BC4 | A | 111 | 18.00 | | 33.39 | | 48.61 | 1M07 | A | 111 | 20.91 | 42.73 | | 36.36 | | 1.8 |
| 1BLQ | A | 90 | 51.07 | | 3.41 | | 45.53 | 1AVS | A | 90 | 62.96 | 4.94 | | 32.10 | | 1.75 |
| 1BLR | A | 137 | 6.55 | | 45.79 | | 47.66 | 2FS6 | A | 137 | 12.50 | 55.15 | | 32.35 | | 1.35 |
| 1BM5 | A | 137 | 12.95 | | 49.93 | | 37.12 | 1XCA | A | 137 | 11.68 | 55.47 | | 32.85 | | 2.3 |
| 1BSH | A | 138 | 22.09 | | 29.10 | | 48.81 | 1FS0 | E | 138 | 27.34 | 41.41 | | 31.25 | | 2.1 |
| 1BU9 | A | 168 | 46.54 | | 0.06 | | 53.41 | 1G3N | B | 168 | 52.60 | 1.30 | | 46.10 | | 2.9 |
| 1CEY | A | 128 | 40.94 | | 15.94 | | 43.12 | 3CHY | A | 128 | 45.31 | 17.19 | | 37.50 | | 1.66 |
| 1D2B | A | 126 | 17.10 | | 28.86 | | 54.04 | 2J0T | D | 126 | 17.74 | 33.06 | | 49.19 | | 2.54 |
| 1DJM | A | 129 | 43.98 | | 16.00 | | 40.02 | 1ZDM | A | 129 | 44.88 | 15.75 | | 39.37 | | 2.4 |
| 1EQ0 | A | 158 | 22.63 | | 17.99 | | 59.39 | 3IP0 | A | 158 | 30.38 | 26.58 | | 43.04 | | 0.89 |
| 1F0Z | A | 66 | 18.91 | | 28.91 | | 52.18 | 1ZUD | A | 66 | 16.92 | 32.31 | | 50.77 | | 1.98 |
| 1F2H | A | 169 | 47.02 | | 15.48 | | 37.50 | 1F3V | A | 179 | 46.50 | 22.29 | | 31.21 | | 2 |
| 1FA4 | A | 105 | 1.95 | | 22.14 | | 75.90 | 2CJ3 | A | 105 | 9.52 | 42.86 | | 47.62 | | 1.7 |
| 1G10 | A | 102 | 20.59 | | 28.43 | | 50.98 | 2BF2 | A | 102 | 29.70 | 37.62 | | 32.67 | | 2.1 |
| 1HFG | A | 71 | 15.71 | | 17.14 | | 67.14 | 2FHT | A | 71 | 17.65 | 26.47 | | 55.88 | | 1.7 |
| 1IDL | A | 74 | 0.55 | | 0.82 | | 98.63 | 1HC9 | B | 74 | 5.41 | 36.49 | | 58.11 | | 1.8 |
| PDB ID  **(NMR)** | Chain | Number of amino acid | Secondary structure diversity (%) | | | | | PDB ID  **(X-ray)** | Chain | Number of amino acid | Secondary structure diversity (%) | | | | | Resolution (Å) |
|  |  |  | Helix | | Beta | | Coil |  |  |  | Helix | Beta | | Coil | |  |
| 1IRY | A | 156 | 14.95 | | 39.53 | | 45.53 | 3ZR0 | A | 156 | 16.34 | 45.10 | | 38.56 | | 1.8 |
| 1IY5 | A | 54 | 20.25 | | 15.85 | | 63.90 | 2OVO | A | 56 | 17.86 | 16.07 | | 66.07 | | 1.5 |
| 1J8I | A | 93 | 16.88 | | 16.94 | | 66.18 | 2NYZ | D | 93 | 22.95 | 24.59 | | 52.46 | | 2.6 |
| 1JOR | A | 149 | 25.77 | | 25.02 | | 49.21 | 1SNO | A | 149 | 26.47 | 33.09 | | 40.44 | | 1.7 |
| 1K19 | A | 112 | 51.08 | | 0.00 | | 48.92 | 1N8V | A | 112 | 67.33 | 0.00 | | 32.67 | | 1.39 |
| 1KJ0 | A | 35 | 0.00 | | 8.53 | | 91.47 | 2F91 | B | 35 | 0.00 | 37.50 | | 62.50 | | 1.2 |
| 1KOT | A | 119 | 31.69 | | 18.47 | | 49.83 | 3D32 | A | 119 | 33.05 | 22.03 | | 44.92 | | 1.3 |
| 1L1I | A | 84 | 0.00 | | 17.89 | | 82.11 | 1EZG | A | 84 | 0.00 | 23.17 | | 76.83 | | 1.4 |
| 1LA3 | A | 201 | 56.08 | | 4.26 | | 39.67 | 1OMV | A | 201 | 64.89 | 4.26 | | 30.85 | | 1.9 |
| 1M9G | A | 97 | 17.55 | | 37.55 | | 44.90 | 3PYJ | A | 97 | 17.89 | 55.79 | | 26.32 | | 2 |
| 1MG8 | A | 78 | 17.40 | | 23.90 | | 58.70 | 2ZEQ | A | 78 | 23.08 | 34.62 | | 42.31 | | 1.65 |
| 1MNL | A | 94 | 18.73 | | 34.38 | | 46.89 | 1MOL | A | 94 | 18.09 | 53.19 | | 28.72 | | 1.7 |
| 1MPH | A | 106 | 17.01 | | 41.54 | | 41.45 | 1BTN | A | 106 | 17.92 | 37.74 | | 44.34 | | 2 |
| 1N88 | A | 96 | 23.59 | | 24.90 | | 51.51 | 3V2D | X | 96 | 24.21 | 36.84 | | 38.95 | | 2.7 |
| 1N9C | A | 71 | 52.91 | | 2.07 | | 45.02 | 1C75 | A | 71 | 52.11 | 2.82 | | 45.07 | | 0.97 |
| 1NSO | A | 107 | 10.19 | | 31.42 | | 58.40 | 3SQF | A | 114 | 10.53 | 36.84 | | 52.63 | | 1.63 |
| 1NVL | A | 86 | 62.73 | | 0.00 | | 37.27 | 1HB6 | A | 86 | 68.60 | 0.00 | | 31.40 | | 2 |
| 1OVQ | A | 138 | 28.00 | | 22.25 | | 49.75 | 1NMN | A | 138 | 34.17 | 33.33 | | 32.50 | | 2.3 |
| 1P4S | A | 181 | 54.89 | | 11.00 | | 34.11 | 2CDN | A | 201 | 54.59 | 12.43 | | 32.97 | | 1.9 |
| 1PFL | A | 139 | 26.85 | | 24.28 | | 48.88 | 2PBD | P | 139 | 28.78 | 31.65 | | 39.57 | | 1.5 |
| 1PUX | A | 124 | 40.85 | | 15.52 | | 43.63 | 1NAT | A | 124 | 47.90 | 22.69 | | 29.41 | | 2.45 |
| PDB ID  **(NMR)** | Chain | Number of amino acid | Secondary structure diversity (%) | | | | | PDB ID  **(X-ray)** | Chain | Number of amino acid | Secondary structure diversity (%) | | | | | Resolution (Å) |
|  |  |  | Helix | Beta | | Coil | |  |  |  | Helix | | Beta | | Coil |  |
| 1Q2N | A | 58 | 67.93 | 0.00 | | 32.07 | | 1LP1 | B | 58 | 70.37 | | 0.00 | | 29.63 | 2.3 |
| 1Q9P | A | 95 | 6.49 | 45.69 | | 47.82 | | 3A2O | A | 99 | 4.04 | | 49.49 | | 46.46 | 0.88 |
| 1R63 | A | 63 | 66.37 | 0.00 | | 33.63 | | 1R69 | A | 69 | 63.49 | | 0.00 | | 36.51 | 2 |
| 1SPY | A | 89 | 57.10 | 5.88 | | 37.02 | | 3SD6 | A | 89 | 60.47 | | 6.98 | | 32.56 | 1.37 |
| 1TL4 | A | 68 | 35.44 | 29.07 | | 35.49 | | 3IWL | A | 68 | 34.85 | | 34.85 | | 30.30 | 1.6 |
| 1TR4 | A | 226 | 51.89 | 0.13 | | 47.98 | | 1UOH | A | 226 | 53.36 | | 0.00 | | 46.64 | 2 |
| 1WKI | A | 141 | 19.29 | 21.10 | | 59.62 | | 3V2D | Q | 141 | 24.11 | | 26.95 | | 48.94 | 2.7 |
| 1WRF | A | 129 | 2.34 | 44.03 | | 53.62 | | 2F08 | A | 129 | 2.33 | | 55.81 | | 41.86 | 2.2 |
| 1YHO | A | 186 | 21.10 | 33.73 | | 45.17 | | 1KMV | A | 186 | 19.89 | | 32.26 | | 47.85 | 1.05 |
| 1ZAC | A | 90 | 67.42 | 6.74 | | 25.84 | | 1AVS | A | 90 | 62.96 | | 4.94 | | 32.10 | 1.75 |
| 2A20 | A | 62 | 10.17 | 25.93 | | 63.90 | | 2CJS | C | 62 | 11.32 | | 33.96 | | 54.72 | 1.78 |
| 2AI5 | A | 80 | 47.56 | 2.50 | | 49.94 | | 1YNR | A | 80 | 51.25 | | 2.50 | | 46.25 | 2 |
| 2CZN | A | 103 | 0.00 | 50.28 | | 49.72 | | 2CWR | A | 103 | 0.00 | | 63.92 | | 36.08 | 1.7 |
| 2DJM | A | 106 | 0.00 | 50.41 | | 49.59 | | 2VQ4 | A | 106 | 2.83 | | 52.83 | | 44.34 | 1.25 |
| 2FKX | A | 88 | 65.07 | 0.13 | | 34.80 | | 1VOQ | O | 88 | 43.18 | | 0.00 | | 56.82 | 11.5 |
| 2GJI | A | 85 | 17.93 | 32.44 | | 49.63 | | 2GJ2 | A | 85 | 16.46 | | 32.91 | | 50.63 | 2.35 |
| 2GPQ | A | 217 | 24.35 | 26.06 | | 49.58 | | 1IPB | A | 217 | 31.41 | | 26.70 | | 41.88 | 2 |
| 2HSY | A | 104 | 33.69 | 20.63 | | 45.68 | | 2FA4 | A | 111 | 39.42 | | 28.85 | | 31.73 | 2.38 |
| 2IGH | A | 61 | 25.00 | 33.82 | | 41.18 | | 1PGX | A | 83 | 20.29 | | 40.58 | | 39.13 | 1.66 |
| 2JN8 | A | 115 | 59.59 | 0.00 | | 40.41 | | 2ES9 | A | 115 | 60.82 | | 0.00 | | 39.18 | 2 |
| 2JR0 | A | 148 | 70.75 | | 0.00 | | 29.25 | 3R2C | A | 148 | 78.99 | 0.00 | | 21.01 | | 1.9 |
| PDB ID  **(NMR)** | Chain | Number of amino acid | Secondary structure diversity (%) | | | | | PDB ID  **(X-ray)** | Chain | Number of amino acid | Secondary structure diversity (%) | | | | | Resolution (Å) |
|  |  |  | Helix | Beta | | Coil | |  |  |  | Helix | | Beta | | Coil |  |
| 2JTM | A | 60 | 0.00 | 43.39 | | 56.61 | | 3LWH | A | 60 | 0.00 | | 42.37 | | 57.63 | 1.9 |
| 2JWT | A | 61 | 64.07 | 0.00 | | 35.93 | | 1HDD | C | 61 | 64.29 | | 0.00 | | 35.71 | 2.8 |
| 2K36 | A | 149 | 54.86 | 0.00 | | 45.14 | | 3JRV | A | 149 | 62.59 | | 0.00 | | 37.41 | 1.6 |
| 2K9C | A | 152 | 19.93 | 1.64 | | 78.42 | | 1Y93 | A | 159 | 24.84 | | 17.20 | | 57.96 | 1.03 |
| 2KFP | A | 125 | 27.90 | 21.49 | | 50.60 | | 3H9X | A | 125 | 28.95 | | 21.93 | | 49.12 | 2.51 |
| 2KLM | A | 147 | 34.86 | 18.36 | | 46.78 | | 3CJT | B | 147 | 30.16 | | 19.05 | | 50.79 | 2.3 |
| 2KQ3 | A | 140 | 24.46 | 24.50 | | 51.04 | | 1SNO | A | 149 | 26.47 | | 33.09 | | 40.44 | 1.7 |
| 2KQY | A | 108 | 36.71 | 2.22 | | 61.06 | | 3FS7 | A | 109 | 54.63 | | 1.85 | | 43.52 | 1.95 |
| 2KSH | A | 110 | 33.05 | 25.14 | | 41.82 | | 1PZ4 | A | 116 | 34.51 | | 28.32 | | 37.17 | 1.35 |
| 2KW2 | A | 101 | 61.15 | 2.00 | | 36.85 | | 3LMO | A | 101 | 64.89 | | 2.13 | | 32.98 | 2 |
| 2KW5 | A | 202 | 26.44 | 23.01 | | 50.55 | | 3MER | A | 202 | 32.95 | | 28.90 | | 38.15 | 2.2 |
| 2L1P | A | 83 | 49.15 | 0.00 | | 50.85 | | 3NZL | A | 83 | 60.00 | | 0.00 | | 40.00 | 1.2 |
| 2L4S | A | 124 | 10.73 | 25.57 | | 63.70 | | 3GJ9 | A | 124 | 14.95 | | 39.25 | | 45.79 | 2.8 |
| 2LCH | A | 113 | 77.01 | 0.00 | | 22.99 | | 3U3B | A | 113 | 82.69 | | 0.00 | | 17.31 | 1.85 |
| 2NO8 | A | 86 | 60.62 | 2.22 | | 37.16 | | 3U43 | A | 94 | 53.26 | | 2.17 | | 44.57 | 1.72 |
| 2OQP | A | 134 | 45.04 | 2.16 | | 52.80 | | 3TGX | B | 134 | 57.39 | | 5.22 | | 37.39 | 2.8 |
| 2P3M | A | 136 | 13.33 | 44.07 | | 42.59 | | 2VBU | A | 136 | 18.32 | | 45.80 | | 35.88 | 1.7 |
| 2RN4 | A | 106 | 4.10 | 44.57 | | 51.33 | | 1JIW | I | 106 | 12.38 | | 51.43 | | 36.19 | 1.74 |
| 2RP3 | A | 101 | 12.65 | 51.10 | | 36.25 | | 3CZZ | A | 101 | 11.88 | | 55.45 | | 32.67 | 1.36 |
| 2RQL | A | 95 | 39.63 | 30.48 | | 29.89 | | 3V26 | X | 101 | 38.95 | | 24.21 | | 36.84 | 3.1 |
| 2Y4W | A | 152 | 34.70 | 15.53 | | 49.77 | | 2YB6 | A | 152 | 35.57 | | 20.13 | | 44.30 | 1.5 |
| PDB ID  **(NMR)** | Chain | Number of amino acid | Secondary structure diversity (%) | | | | | PDB ID  **(X-ray)** | Chain | Number of amino acid | Secondary structure diversity (%) | | | | | Resolution (Å) |
|  |  |  | Helix | Beta | | Coil | |  |  |  | Helix | | Beta | | Coil |  |
| 2YUV | A | 100 | 0.30 | 48.65 | | 51.05 | | 2YXM | A | 100 | 7.61 | | 53.26 | | 39.13 | 1.51 |
| 3GB1 | A | 56 | 25.51 | 40.00 | | 34.49 | | 1PGB | A | 56 | 25.00 | | 42.86 | | 32.14 | 1.92 |
