# Supplementary material for: Protein NMR Structures Refined without NOE Data
Source: PLoS One. 2014 Oct 3;9(10):e108888. doi: 10.1371/journal.pone.0108888 (PMC4184813; doi:10.1371/journal.pone.0108888)
Supplement: Table S3 — Various scores and their weights for the normalized score. (DOCX) [file pone.0108888.s005.docx]

Table S3. Various scores and their weights for the normalized score

| *i* | Scores | Bad (*b_i_*) | Good (g*_i_*) | Weight1 | Weight2 |
| --- | --- | --- | --- | --- | --- |
| 1 | TM-score | 0.4 | 1 | 1 | 0 |
| 2 | NOE violation | 1 | 0 | 1 | 0 |
| 3 | nDOPE | 0 | -2 | 0.1 | 0.1 |
| 4 | dDFIRE | 0 | -500 | 0.1 | 0.1 |
| 5 | Clash | 10 | 0 | 0.1 | 0.1 |
| 6 | Rama (MOL) | 70 | 100 | 0.1 | 0.1 |
| 7 | Rama (PRO) | 70 | 100 | 0.1 | 0.1 |
| 8 | 1^st^ Packing | -2 | 2 | 0.1 | 0.1 |
| 9 | 2^nd^ Packing | -2 | 2 | 0.1 | 0.1 |
| 10 | Rama (WHAT) | -2 | 2 | 0.1 | 0.1 |
| 11 | Rotamer | -2 | 2 | 0.1 | 0.1 |
| 12 | Backbone | -2 | 2 | 0.1 | 0.1 |
