# Supplementary material for: Protein NMR Structures Refined without NOE Data
Source: PLoS One. 2014 Oct 3;9(10):e108888. doi: 10.1371/journal.pone.0108888 (PMC4184813; doi:10.1371/journal.pone.0108888)
Supplement: Table S4 — Quality assessment scores and total score in S1 2,000 step. (DOCX) [file pone.0108888.s006.docx]

Table S4. Quality assessment scores and total score in *S1* 2,000 step

| Distance  width | TM-score^b^ | NOE violation | DOPE | nDOPE | dDFIRE | Clash | Rama  (MOL) | Rama  (PRO) | 1st  packing | 2nd  packing | Rama  (WHAT) | Rotamer | Backbone | Total  score |
| --- | --- | --- | --- | --- | --- | --- | --- | --- | --- | --- | --- | --- | --- | --- |
| 0 | 0.788 | 0.539 | -5712.60 | 1.1804 | -111.825 | 1.49 | 88.30 | 80.10 | -7.183 | -4.237 | -2.930 | -4.597 | -4.060 | 0.926 |
| 1 | 0.775 | 0.536 | -5975.05 | 1.0097 | -117.305 | 0.67 | 90.50 | 83.17 | -7.257 | -4.385 | -1.885 | -3.387 | -3.759 | 1.000 |
| 2 | 0.762 | 0.546 | -6433.85 | 0.6933 | -126.394 | 0.35 | 92.52 | 86.18 | -7.058 | -4.578 | -0.891 | -1.965 | -3.409 | 1.076 |
| 3 | 0.764 | 0.546 | -7021.46 | 0.3111 | -141.695 | 0.18 | 94.08 | 88.75 | -6.284 | -4.31 | 0.088 | -0.411 | -2.673 | 1.225 |
| 4 | 0.776 | 0.534 | -7763.53 | -0.1580 | -160.676 | 0.15 | 95.25 | 90.63 | -5.055 | -3.351 | 0.974 | 0.900 | -1.735 | 1.428 |
| 5 | 0.777 | 0.527 | -8404.12 | -0.5466 | -175.872 | 0.14 | 95.84 | 91.80 | -4.041 | -2.516 | 1.558 | 1.698 | -1.233 | 1.559 |
| 6^a^ | 0.767 | 0.506 | -8773.02 | -0.7630 | -184.412 | 0.16 | 96.08 | 92.33 | -3.484 | -2.066 | 1.798 | 2.097 | -1.035 | 1.624 |
| 7 | 0.753 | 0.526 | -8945.64 | -0.8630 | -187.843 | 0.18 | 96.09 | 92.54 | -3.232 | -1.88 | 1.926 | 2.276 | -0.965 | 1.607 |
| 8 | 0.737 | 0.558 | -8984.96 | -0.8854 | -188.571 | 0.19 | 96.21 | 92.70 | -3.152 | -1.82 | 1.983 | 2.369 | -0.932 | 1.558 |
| 9 | 0.721 | 0.596 | -8961.24 | -0.8682 | -188.116 | 0.20 | 96.27 | 92.86 | -3.175 | -1.842 | 2.017 | 2.410 | -0.923 | 1.495 |
| 10 | 0.707 | 0.632 | -8911.22 | -0.8354 | -187.181 | 0.20 | 96.31 | 92.90 | -3.222 | -1.882 | 2.028 | 2.421 | -0.914 | 1.431 |

^a^ Shadowed line indicate the optimal width

^b^ The NMR original structure was used for reference structure of TM-score.
