# Supplementary material for: Protein NMR Structures Refined without NOE Data
Source: PLoS One. 2014 Oct 3;9(10):e108888. doi: 10.1371/journal.pone.0108888 (PMC4184813; doi:10.1371/journal.pone.0108888)
Supplement: Table S5 — Quality assessment scores and total score in S1 5,000 step. (DOCX) [file pone.0108888.s007.docx]

| Distance  width | TM-score^b^ | NOE violation | DOPE | nDOPE | dDFIRE | Clash | Rama  (MOL) | Rama  (PRO) | 1st packing | 2nd packing | Rama  (WHAT) | Rotamer | Backbone | Total  score |
| --- | --- | --- | --- | --- | --- | --- | --- | --- | --- | --- | --- | --- | --- | --- |
| 0 | 0.785 | 0.511 | -5714.36 | 1.1942 | -113.596 | 1.44 | 88.62 | 80.51 | -7.17836 | -4.23862 | -2.87583 | -4.57315 | -4.06549 | 0.9528 |
| 1 | 0.772 | 0.506 | -5976.76 | 1.0067 | -117.649 | 0.65 | 90.52 | 83.18 | -7.25058 | -4.38737 | -1.87999 | -3.38509 | -3.75259 | 1.0272 |
| 2 | 0.759 | 0.516 | -6436.66 | 0.6897 | -126.79 | 0.35 | 92.54 | 86.25 | -7.04873 | -4.576 | -0.876356 | -1.93224 | -3.40024 | 1.1038 |
| 3 | 0.762 | 0.519 | -7027.55 | 0.3056 | -142.255 | 0.19 | 94.13 | 88.82 | -6.27116 | -4.29831 | 0.114112 | -0.333399 | -2.66328 | 1.2515 |
| 4 | 0.774 | 0.507 | -7773.77 | -0.1655 | -161.397 | 0.13 | 95.31 | 90.78 | -5.04012 | -3.32557 | 0.99602 | 1.04081 | -1.73447 | 1.4584 |
| 5 | 0.775 | 0.502 | -8415.34 | -0.5530 | -176.689 | 0.14 | 95.92 | 91.96 | -4.03071 | -2.48222 | 1.57452 | 1.8916 | -1.23132 | 1.5872 |
| 6^a^ | 0.764 | 0.508 | -8791.50 | -0.7760 | -184.883 | 0.15 | 96.20 | 92.43 | -3.45921 | -2.0183 | 1.83827 | 2.30987 | -1.01749 | 1.6275 |
| 7 | 0.749 | 0.531 | -8968.07 | -0.8776 | -188.437 | 0.18 | 96.29 | 92.80 | -3.19939 | -1.81384 | 1.9679 | 2.50606 | -0.926745 | 1.6086 |
| 8 | 0.732 | 0.564 | -9015.94 | -0.9028 | -189.389 | 0.18 | 96.39 | 93.09 | -3.11793 | -1.76108 | 2.01859 | 2.59008 | -0.895401 | 1.5565 |
| 9 | 0.715 | 0.602 | -8986.94 | -0.8838 | -188.849 | 0.19 | 96.39 | 93.14 | -3.14455 | -1.78497 | 2.07056 | 2.63553 | -0.884807 | 1.4911 |
| 10 | 0.700 | 0.642 | -8932.97 | -0.8503 | -187.852 | 0.19 | 96.48 | 93.34 | -3.19251 | -1.81625 | 2.10172 | 2.66021 | -0.861014 | 1.4246 |

Table S5. Quality assessment scores and total score in *S1* 5,000 step

^a^ Shadowed line indicate the optimal width

^b^ The NMR original structure was used for reference structure of TM-score.
