# Supplementary material for: Protein NMR Structures Refined without NOE Data
Source: PLoS One. 2014 Oct 3;9(10):e108888. doi: 10.1371/journal.pone.0108888 (PMC4184813; doi:10.1371/journal.pone.0108888)
Supplement: Table S6 — Quality assessment scores and total score in S1 10,000 step. (DOCX) [file pone.0108888.s008.docx]

Table S6. Quality assessment scores and total score in *S1* 10,000 step

| Distance  width | TM-score^b^ | NOE violation | DOPE | nDOPE | dDFIRE | Clash | Rama  (MOL) | Rama  (PRO) | 1st  packing | 2nd packing | Rama  (WHAT) | Rotamer | Backbone | Total  score |
| --- | --- | --- | --- | --- | --- | --- | --- | --- | --- | --- | --- | --- | --- | --- |
| 0 | 0.782 | 0.512 | -5715.94 | 1.19321 | -113.584 | 1.44 | 88.63 | 80.53 | -7.17307 | -4.2480 | -2.87267 | -4.57926 | -4.059 | 0.948 |
| 1 | 0.769 | 0.507 | -5978.55 | 1.00568 | -117.643 | 0.66 | 90.51 | 83.19 | -7.24609 | -4.39416 | -1.87595 | -3.38461 | -3.75198 | 1.019 |
| 2 | 0.755 | 0.517 | -6440.12 | 0.687662 | -126.868 | 0.34 | 92.59 | 86.24 | -7.04396 | -4.57327 | -0.858598 | -1.9037 | -3.38927 | 1.097 |
| 3 | 0.756 | 0.521 | -7027.53 | 0.305226 | -142.253 | 0.19 | 94.24 | 88.92 | -6.27174 | -4.29688 | 0.131432 | -0.255821 | -2.6674 | 1.244 |
| 4 | 0.769 | 0.512 | -7775.55 | -0.168522 | -161.472 | 0.13 | 95.46 | 90.92 | -5.03272 | -3.31186 | 1.03131 | 1.16535 | -1.72635 | 1.450 |
| 5 | 0.770 | 0.503 | -8422.35 | -0.560608 | -176.988 | 0.13 | 96.02 | 92.14 | -4.00762 | -2.44694 | 1.60006 | 2.05592 | -1.21535 | 1.586 |
| 6 ^a^ | 0.760 | 0.512 | -8811.47 | -0.788015 | -185.452 | 0.15 | 96.33 | 92.67 | -3.43084 | -1.97747 | 1.88083 | 2.48126 | -0.99804 | 1.625 |
| 7 | 0.744 | 0.534 | -8992.66 | -0.893989 | -189.14 | 0.17 | 96.42 | 93.05 | -3.15784 | -1.76674 | 2.01683 | 2.6964 | -0.903518 | 1.607 |
| 8 | 0.727 | 0.567 | -9042.53 | -0.920782 | -190.06 | 0.19 | 96.49 | 93.26 | -3.08811 | -1.71884 | 2.06345 | 2.78008 | -0.870883 | 1.556 |
| 9 | 0.709 | 0.604 | -9025.3 | -0.909022 | -189.766 | 0.19 | 96.51 | 93.43 | -3.09719 | -1.72212 | 2.09452 | 2.81371 | -0.857804 | 1.490 |
| 10 | 0.693 | 0.649 | -8968.42 | -0.872825 | -188.733 | 0.19 | 96.52 | 93.50 | -3.14905 | -1.76799 | 2.12415 | 2.83812 | -0.842199 | 1.416 |

^a^ Shadowed line indicate the optimal width

^b^ The NMR original structure was used for reference structure of TM-score.
