# Supplementary material for: Protein NMR Structures Refined without NOE Data
Source: PLoS One. 2014 Oct 3;9(10):e108888. doi: 10.1371/journal.pone.0108888 (PMC4184813; doi:10.1371/journal.pone.0108888)
Supplement: Table S7 — Quality assessment scores and total score in S2. (DOCX) [file pone.0108888.s009.docx]

Table S7. Quality assessment scores and total score in *S2*

| Distance width | TM-score^b^ | NOE violation | DOPE | nDOPE | dDFIRE | Clash | Rama  (MOL) | Rama  (PRO) | 1st  packing | 2nd packing | Rama  (WHAT) | Rotamer | Backbone | Total score |
| --- | --- | --- | --- | --- | --- | --- | --- | --- | --- | --- | --- | --- | --- | --- |
| 0 | 0.989 | 0.242343 | -8844.07 | -0.7581 | -180.176 | 12.61 | 89.02 | 80.81 | -3.2413 | -2.5399 | -2.3825 | -3.99626 | -1.15604 | 1.8034 |
| 1 | 0.955 | 0.276 | -8987.8 | -0.8638 | -184.578 | 2.11 | 92.42 | 85.71 | -3.2008 | -2.2842 | -0.3396 | -1.7552 | -1.0663 | 1.9690 |
| 2 ^a^ | 0.920 | 0.306 | -9064.56 | -0.9172 | -187.151 | 0.89 | 93.92 | 88.08 | -3.1283 | -2.1078 | 0.5401 | -0.402042 | -1.04533 | 1.9717 |
| 3 | 0.888 | 0.334 | -9115.46 | -0.9522 | -188.887 | 0.532 | 94.81 | 89.60 | -3.0642 | -1.9831 | 1.0237 | 0.458744 | -1.03826 | 1.9429 |
| 4 | 0.861 | 0.359 | -9150.96 | -0.9762 | -190.082 | 0.39 | 95.38 | 90.55 | -3.0196 | -1.8789 | 1.3136 | 1.01103 | -1.01993 | 1.9047 |
| 5 | 0.837 | 0.385 | -9165.86 | -0.9873 | -190.728 | 0.31 | 95.69 | 91.17 | -2.9960 | -1.8267 | 1.5043 | 1.39883 | -1.00532 | 1.8609 |
| 6 | 0.818 | 0.410 | -9167.97 | -0.9908 | -191.117 | 0.26 | 95.89 | 91.68 | -2.9806 | -1.7791 | 1.6554 | 1.70615 | -0.975983 | 1.8219 |
| 7 | 0.800 | 0.435 | -9155.68 | -0.9830 | -191.123 | 0.23 | 95.97 | 91.99 | -2.9880 | -1.7603 | 1.7489 | 1.91557 | -0.973906 | 1.7758 |
| 8 | 0.785 | 0.460 | -9135.87 | -0.9708 | -190.913 | 0.23 | 96.07 | 92.28 | -3.0026 | -1.7531 | 1.8140 | 2.04889 | -0.957566 | 1.7305 |
| 9 | 0.770 | 0.485 | -9105.27 | -0.9523 | -190.498 | 0.22 | 96.13 | 92.42 | -3.0307 | -1.7631 | 1.8775 | 2.16507 | -0.932379 | 1.6853 |
| 10 | 0.757 | 0.514 | -9072.94 | -0.9330 | -189.994 | 0.21 | 96.18 | 92.6344 | -3.0622 | -1.7858 | 1.9373 | 2.23896 | -0.922676 | 1.6363 |

^a^ Shadowed line indicate the optimal width

^b^ The NMR original structure was used for reference structure of TM-score.
