# Supplementary material for: Protein NMR Structures Refined without NOE Data
Source: PLoS One. 2014 Oct 3;9(10):e108888. doi: 10.1371/journal.pone.0108888 (PMC4184813; doi:10.1371/journal.pone.0108888)
Supplement: Table S8 — Quality assessment scores in 50 optimization set. (DOCX) [file pone.0108888.s010.docx]

Table S8. Quality assessment scores in 50 optimization set

| width | TM-score^b^ | NOE violation | nDOPE | dDFIRE | Clash | Rama  (MOL) | Rama  (PRO) | 1st packing | 2^nd^  packing | Rama  (what) | Rotomer  (what) | Backbone  (what) | Total  score |
| --- | --- | --- | --- | --- | --- | --- | --- | --- | --- | --- | --- | --- | --- |
| 0 | 0.75 | 0.268 | -1.0764 | -189.3 | 14.07 | 89.03 | 80.87 | -2.570 | -2.216 | -2.224 | -3.853 | -1.172 | 1.417 |
| 1 | 0.76 | 0.299 | -1.1567 | -193.141 | 2.25 | 92.38 | 85.59 | -2.602 | -2.024 | -0.213 | -1.680 | -1.082 | 1.655 |
| 2 | 0.76 | 0.326 | -1.1961 | -195.259 | 0.84 | 93.79 | 87.51 | -2.568 | -1.860 | 0.706 | -0.406 | -1.066 | 1.719 |
| 3 | 0.76 | 0.350 | -1.2259 | -196.818 | 0.50 | 94.87 | 89.19 | -2.500 | -1.738 | 1.197 | 0.459 | -1.056 | 1.749 |
| 4 ^a^ | 0.76 | 0.371 | -1.2464 | -198.008 | 0.35 | 95.48 | 90.09 | -2.443 | -1.664 | 1.528 | 1.024 | -1.028 | 1.764 |
| 5 | 0.76 | 0.391 | -1.2502 | -198.489 | 0.28 | 95.61 | 90.84 | -2.419 | -1.638 | 1.725 | 1.390 | -1.064 | 1.760 |
| 6 | 0.76 | 0.411 | -1.2601 | -199.193 | 0.19 | 96.02 | 91.20 | -2.389 | -1.549 | 1.825 | 1.747 | -1.023 | 1.756 |
| 7 | 0.75 | 0.431 | -1.2517 | -199.208 | 0.14 | 96.03 | 91.67 | -2.395 | -1.552 | 1.902 | 1.988 | -1.016 | 1.738 |
| 8 | 0.75 | 0.447 | -1.2354 | -198.952 | 0.16 | 96.17 | 91.96 | -2.408 | -1.523 | 1.983 | 2.110 | -1.013 | 1.720 |
| 9 | 0.74 | 0.471 | -1.2120 | -198.563 | 0.15 | 96.21 | 92.19 | -2.426 | -1.495 | 2.020 | 2.311 | -1.002 | 1.693 |
| 10 | 0.73 | 0.492 | -1.1887 | -198.01 | 0.13 | 96.34 | 92.36 | -2.478 | -1.529 | 2.060 | 2.358 | -1.002 | 1.661 |

^a^ Shadowed line indicate the optimal width

^b^ The NMR original structure was used for reference structure of TM-score.
