# Supplementary material for: Protein NMR Structures Refined without NOE Data
Source: PLoS One. 2014 Oct 3;9(10):e108888. doi: 10.1371/journal.pone.0108888 (PMC4184813; doi:10.1371/journal.pone.0108888)
Supplement: Table S9 — PDB list of AMBER or RECOORD comparison set. (DOCX) [file pone.0108888.s011.docx]

Table S9. PDB list of AMBER or RECOORD comparison set

| PDB  (NMR) | PDB  (X-ray) | PDB  (NMR) | PDB  (X-ray) |
| --- | --- | --- | --- |
| 1CN7 | 1NMU | 2HGM | - |
| 1R4Y | - | 2HRF | - |
| 1S6D | - | 2K3X | - |
| 1SB6 | 2XMJ | 2K4W | - |
| 1T0W | - | 2K6V | - |
| 1V3A | - | 2KCF | - |
| 1WT8 | - | 2KUQ | - |
| 1YSM | - | 2L3N | - |
| 2AJ0 | - | 2L4O | - |
| 2DCO | - | 2RLK | - |
| 2DDY | - | 2RN9 | - |
| 2GL1 | - |  |  |
